# Tissue engineering strategies to bioengineer the ageing skin phenotype in vitro

**DOI:** 10.1111/acel.13550

**Published:** 2022-01-17

**Authors:** Lydia Costello, Teresa Dicolandrea, Ryan Tasseff, Robert Isfort, Charlie Bascom, Thomas von Zglinicki, Stefan Przyborski

**Affiliations:** ^1^ Department of Biosciences Durham University Durham UK; ^2^ Procter and Gamble Mason Business Center Cincinnati Ohio USA; ^3^ Institute for Cell and Molecular Sciences Newcastle University Newcastle Upon Tyne UK; ^4^ Reprocell Europe Glasgow, Durham UK

**Keywords:** ageing, bioengineered tissue, human, in vitro, molecular biology of aging, skin

## Abstract

Human skin ageing is a complex and heterogeneous process, which is influenced by genetically determined intrinsic factors and accelerated by cumulative exposure to extrinsic stressors. In the current world ageing demographic, there is a requirement for a bioengineered ageing skin model, to further the understanding of the intricate molecular mechanisms of skin ageing, and provide a distinct and biologically relevant platform for testing actives and formulations. There have been many recent advances in the development of skin models that recapitulate aspects of the ageing phenotype in vitro. This review encompasses the features of skin ageing, the molecular mechanisms that drive the ageing phenotype, and tissue engineering strategies that have been utilised to bioengineer ageing skin in vitro.

## Graphical abstract

In the world ageing population, there is a demand for bioengineered ageing skin models, to further understanding of the molecular mechanisms of skin ageing and provide a predictive in vitro platform for testing of anti‐ageing actives. This review explores the features of ageing skin, molecular mechanisms that drive the ageing skin phenotype, and discusses how tissue engineering strategies can be applied to model this phenomenon in vitro.

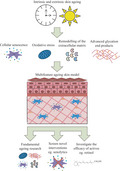

## 1 INTRODUCTION

**FIGURE 1:**
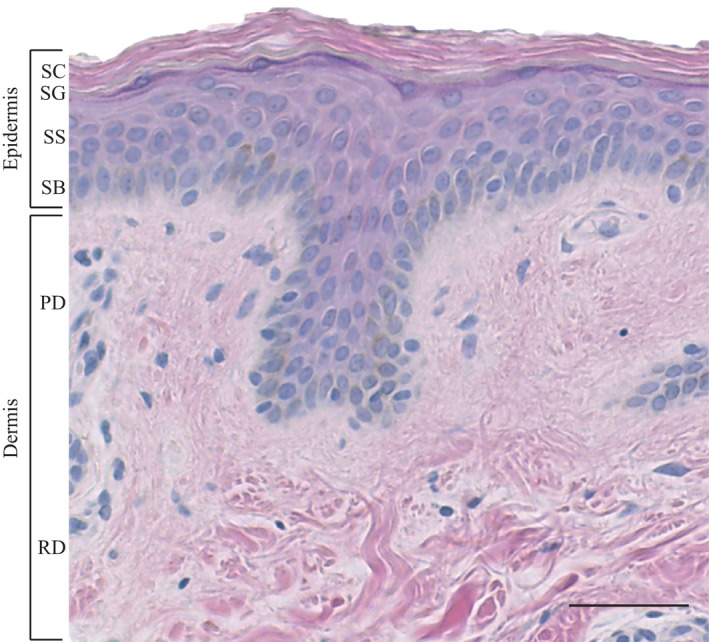
Human skin architecture. Human skin is composed of the epidermis, dermis and hypodermis. The epidermal layers include the *stratum basale* (SB), *stratum spinosum* (SS), *stratum granulosum* (SG) and *stratum corneum* (SC). The dermis is composed of the papillary (PD) and reticular (RD) layers. Scale bar: 50 μm

The skin is the largest organ in the human body, with a surface area of approximately 2 m^2^ (Mosteller, 1987). It has a multicellular and multi‐layered topographical structure, with a complex microenvironment enriched in spatiotemporal chemical and mechanical cues (Figure 1). Due to its direct contact with the external environment, the skin has multiple specialised functions including thermoregulation, immunological surveillance and the provision of a protective barrier against external chemical, mechanical and pathogenic insults. Similar to all other organs in the human body, the skin undergoes a structural and functional decline with age.

Skin ageing is a multifactorial process, which manifests as cumulative and progressive changes in the structure of human skin. These age‐dependent morphological alterations can lead to many debilitating skin conditions in the elderly, such as xerosis cutis, pruritus and defective wound healing. Most individuals over 65 years’ experience two or more dermatological conditions that require medical attention (Kligman & Koblenzer, 1997). In addition to cutaneous disorders, as the skin is the most visible indicator of age, phenotypic changes in physical appearance such as pronounced wrinkles, increased laxity and irregular pigmentation often have many psychosocial implications such as reduced self‐esteem (Farage et al., 2010). This drives an enormous consumer demand for anti‐ageing treatments and the development of cosmetic interventions.

Population ageing is a global phenomenon, and the number of individuals aged 60 years or over is projected to increase by 56% to 1.4 billion by 2030 (United Nations, 2015). This demographic shift towards an ageing population suggests that dermatological studies should focus on informing strategies to alleviate the deleterious effects of skin ageing and age‐related dermatoses. Bioengineered skin constructs recapitulate the complex structure of human skin in vitro and provide a physiologically relevant platform for skin ageing research. In this article, we will review the features of ageing skin, the mechanisms that drive the ageing process, and how ageing skin can be bioengineered in vitro as a research tool.

## 2 IMPACT OF AGEING ON SKIN ANATOMY AND PHYSIOLOGY

**FIGURE 2:**
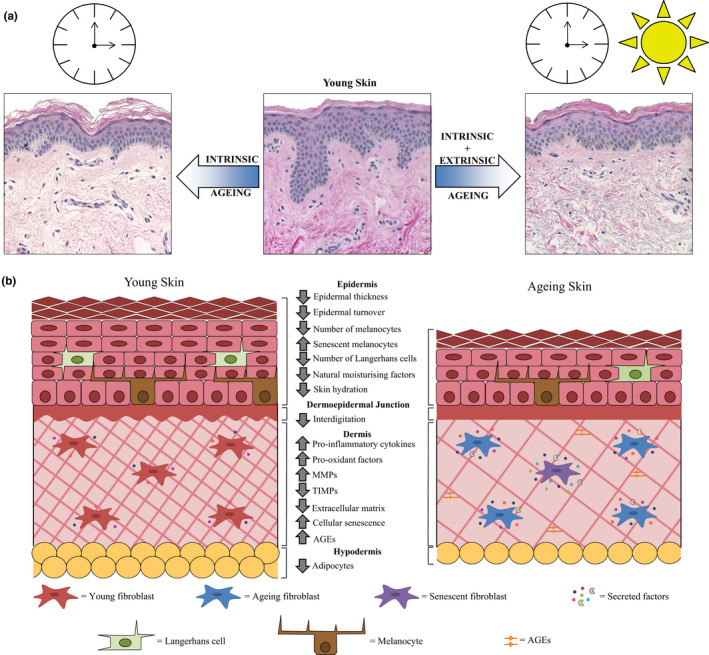
Age‐related changes in human skin. (a) Human skin undergoes distinct remodelling with age, as a result of intrinsic changes and extrinsic lifestyle factors. The young skin sample is from a photoprotected site of a 21‐year‐old female, the intrinsically aged skin sample is from a photoprotected site of a 64‐year‐old female, and the extrinsically aged skin sample is from a photoexposed site of a 65‐year‐old female. (b) Many characteristic structural changes are observed in the epidermis, dermis and hypodermis with age, which contribute to the ageing skin phenotype

Human skin has a composite organisation composed of three layers: the epidermis, dermis and hypodermis, with distinct structures and functions. The clinical features of ageing skin are attributed to age‐related alterations in the different strata (Figure 2). An understanding of these anatomical changes is important to ensure that tissue‐engineered skin models successfully recreate aspects of the ageing skin phenotype in vitro.

### 2.1 Epidermis

The epidermis has a differentiated, stratified and keratinised structure, with a multitude of cell types including immunoregulatory Langerhans cells, photoprotective melanocytes and keratinocytes, which predominantly constitute 95% of the epidermis (Brenner & Hearing, 2008; Streilein & Bergstresser, 1984). The proliferative keratinocytes in the *stratum basale* undergo profound morphological, cytoarchitectural and lipogenic changes during sequential differentiation to form the suprabasal *stratum spinosum* and *stratum granulosum* layers (Eckert & Rorket, 1989). Keratinocytes then undergo terminal differentiation to form corneocytes within the lipid‐rich, proteinaceous *stratum corneum*.

There are many structural and functional changes that occur in the epidermis with age. The stem cell functional capacity is impaired in the ageing epidermis, with a reduced mitotic activity of keratinocytes in the *stratum basale*, and a decreased epidermal turnover rate of approximately 30% to 50% by 80 years of age (Grove & Kligman, 1983). This is associated with an atrophic phenotype, and a decreased epidermal thickness of approximately 6.4% per decade (Branchet et al., 1990). In addition to age‐related changes in keratinocytes, the number of melanocytes decreases from 8 to 20% per decade whilst the number of senescent melanocytes increases with age and contributes to the pallor of ageing skin and loss of hair pigmentation (Gilchrest et al., 1979; Quevedo et al., 1969; Victorelli et al., 2019). In chronically photoexposed skin, hyperpigmented macules known as senile lentigo are observed, which are characterised by elongated rête ridges and an increase in the quantity and activity of melanocytes (Hodgson, 1963). The number of immunoregulatory Langerhans cells also decreases with age in sun‐exposed skin, by approximately 50% by 80 years, which affects skin immunity (Gilchrest et al., 1983).

The ageing epidermis is thought to have a compromised barrier, which contributes to the clinical manifestation of abnormally dry skin. Skin hydration decreases with age due to the reduction of hygroscopic natural moisturising factors, diminished lipid‐processing enzymes and an altered lipid composition in the *stratum corneum* (Jacobson et al., 1990; Rogers et al., 1996; Starr et al., 2016). Alterations in sebaceous glands also contribute to the xerotic phenotype, as although the quantity is not affected by age, a reduction in cellular turnover results in hyperplasia of facial glands, and the secretion of sebum is reduced by almost 40% (Zouboulis & Boschnakow, 2001).

### 2.2 Dermoepidermal junction

The epidermis is adhered to the dermis via the interdigitating dermoepidermal junction (DEJ), which facilitates the regulated exchange of substances and aids polarity of basal keratinocytes (Briggaman & Wheeler, 1975). The integrity of the DEJ decreases with age, and in ageing skin, the quantity of fundamental components such as collagen IV, collagen VII and laminin‐332 is reduced (Langton et al., 2016). The DEJ also undergoes a characteristic flattening with age, and the number of dermal papillae and height of rête ridges is significantly decreased (Montagna & Carlisle, 1979; Sauermann et al., 2002). The surface area of the DEJ decreases by approximately 35%, which reduces cellular communication and the transfer of nutrients, oxygen and growth factors to the avascular epidermis (Moragas et al., 1993).

### 2.3 Dermis

The human dermis has a dynamic architecture, containing a multitude of cell types such as fibroblasts and immune cells, and a composite network of extracellular matrix (ECM) proteins, which provide the skin with mechanical strength and elastic recoil. Structurally, it is composed of two morphologically distinct layers: the superficial papillary dermis containing sparsely arranged collagen, oxytalan and elaunin fibres orientated perpendicularly to the DEJ, and the reticular dermis containing abundant collagen fibres and a dense elastic network (Cotta‐Pereira et al., 1976; Meigel et al., 1977). The fibrous proteins are embedded in an amorphous matrix of hydrophilic proteoglycans, glycoproteins and glycosaminoglycans, which provide hydration, turgidity and viscoelastic properties to withstand external compression forces.

The dermis undergoes prominent structural alterations with age, and there is a decline in dermal integrity correlated with reduced skin elasticity, extensibility and increased laxity. As fibroblasts age, the secretome undergoes compositional changes, with an upregulation of pro‐inflammatory cytokines, pro‐oxidant factors and degradative matrix metalloproteinases (MMPs) (Waldera Lupa et al., 2015). The increase in MMP secretion contributes to one of the most prominent features of dermal ageing: atrophy of the extracellular matrix networks. The chronic degradation of the fibrous proteins and amorphous matrix alters tissue biomechanics with age (Fisher et al., 1997). The fragmentation of the extracellular matrix reduces the mechanical tension exerted on the dermal fibroblasts. This causes fibroblasts to collapse with age, which results in a further reduction of collagen synthesis and higher levels of MMPs, and exacerbates the dermal atrophy in a self‐perpetuating cycle (Fisher et al., 2008). In contrast, in photoaged skin, there is an abnormal accumulation of disorganised, amorphous elastin fragments, known as actinic elastosis (Sellheyer, 2003).

### 2.4 Hypodermis

The hypodermis consists of lobular adipose tissue, which connects the skin to the underlying fascia. It plays a vital role in fat storage, thermoregulation and shock absorption of external trauma. Ageing is associated with atrophy of the hypodermis, which reduces trauma resistance, and contributes to the wrinkling and sagging of skin (Piérard et al., 2003).

## 3 INTRINSIC AND EXTRINSIC SKIN AGEING

The structural and functional changes that occur in the skin with age can be attributed to the synergistic and deleterious consequences of intrinsic and extrinsic factors. There are anatomical variations in skin ageing, as morphological changes in photoprotected sites are mainly caused by intrinsic factors, whereas photoexposed sites have an accelerated ageing phenotype. In most sites, it is difficult to distinguish the respective contributions of intrinsic and extrinsic factors to the ageing phenotype.

### 3.1 Intrinsic skin ageing

Intrinsic ageing is an inherent, genetically determined phenomenon that occurs in all organs with age due to the accumulation of molecular damage. Intrinsically aged skin typically has a thin, uniformly pigmented complexion with fine lines and wrinkles (Durai et al., 2012). The rate of intrinsic skin ageing is associated with factors such as ethnicity, metabolism and hormonal changes, which, in females, includes a decrease in oestrogen during menopause (van Beek et al., 2016; Thornton, 2013; Vashi et al., 2016).

### 3.2 Extrinsic skin ageing

In addition to the inevitable, chronological decline, the skin also undergoes extrinsic ageing due to its interface with the environment. The clinical features of extrinsically aged skin include pronounced deep wrinkles, dyspigmentation and increased laxity, particularly in areas of dynamic facial expression such as the periorbital and periocular regions (Durai et al., 2012). The pathophysiological changes in extrinsically aged skin are attributed to environmental factors such as ultraviolet (UV) radiation; and lifestyle factors such as diet, health status and smoking (Krutmann et al., 2017). Chronic sun exposure is thought to be a prominent contributor to the extrinsically aged skin phenotype, and in Caucasian individuals, UV radiation contributes to approximately 80% of facial ageing (Flament et al., 2013). Chronic exposure to UV radiation also induces photodamage, which increases the risk of benign and malignant cutaneous neoplasms (Narayanan et al., 2010).

The morphological effects of extrinsic skin ageing are superimposed on intrinsic ageing, which exacerbates the accumulation of pathophysiological changes to cutaneous cells and structural alterations in the human dermis.

## 4 MOLECULAR MECHANISMS OF SKIN AGEING

**FIGURE 3:**
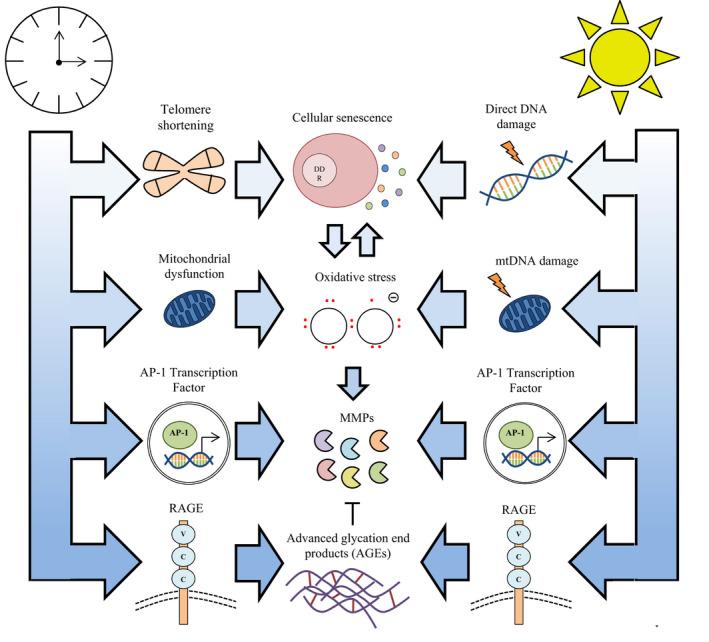
Molecular mechanisms of skin ageing. Intrinsic and extrinsic skin ageing have overlapping, underpinning molecular mechanisms such as cellular senescence, oxidative stress, the upregulation of MMPs and deposition of AGEs that drive the altered phenotype

Although some clinical features of intrinsic and extrinsic skin ageing are phenotypically distinct, there are several interconnected molecular similarities that encompass the nine hallmarks of ageing (Lopez‐Otin et al., 2013). These factors contribute to skin ageing by inducing oxidative stress, remodelling of the extracellular matrix and the deposition of advanced glycation end products (AGEs) within the dermis (Figure 3). The underlying mechanisms that drive human skin ageing have been used to bioengineer ageing skin models in vitro, for example, through the incorporation of senescent cells or advanced glycation end products, which is discussed in further detail later in this review.

### 4.1 Cellular senescence

Cellular senescence is defined as an irreversible loss of mitotic activity associated with major changes in the cellular phenotype including induction of a senescence‐associated secretory phenotype (SASP) and mitochondrial dysfunction (Gorgoulis et al., 2019). The induction of cellular senescence is frequently initiated through a persistent DNA damage response, and the activation of ataxia telangiectasia mutated (ATM) and ATM‐ and Rad‐3‐related (ATR) kinases. Downstream signalling pathways activate p53 to elicit cell‐cycle arrest and a senescent phenotype. The induction of cellular senescence may also involve the retinoblastoma (Rb) pathway, and the relative contributions of the p53‐p21 and p16‐Rb pathways depend on the type of stressor involved (Campisi & d’Adda di Fagagna, 2007; Van Deursen, 2014).

The complex, multi‐step initiation of cellular senescence is associated with remodelling of its higher‐order chromatin, and the formation of punctate facultative heterochromatin, known as senescence‐associated heterochromatin foci. These chromatin alterations cause profound transcriptional changes, which alter cellular function and modulate the microenvironment through secretory factors (Shelton et al., 1999). This characteristic secretome of pro‐inflammatory cytokines and chemokines, pro‐oxidant reactive oxygen species and degradative proteases is collectively known as the SASP (Coppé et al., 2008; Nelson et al., 2012).

The accumulation of senescent cells with age has been proposed to accelerate organismal ageing. Several proof‐of‐principle experiments have demonstrated that the selective elimination of senescent cells in mouse models increased lifespan and reduced age‐related pathologies (van Beek et al., 2016; Xu et al., 2018). There is an age‐dependent increase in senescent cells in both the epidermis and dermis with age, which is thought to contribute to the age‐related decline in skin homeostasis (Dimri et al., 1995; Ressler et al., 2006; Victorelli et al., 2019).

#### 4.1.1 Cellular senescence in the epidermis

Although the skin accumulates senescent cells with age, it remains controversial whether epidermal stem cells age, as they are responsible for the crucial maintenance of the epidermal barrier throughout an individual's lifetime. A study using organotypic models found that the overexpression of p16^INK4a^ in young keratinocytes resulted in epidermal atrophy, which suggests that cellular senescence could play a causal role in the ageing epidermal phenotype; however, the number of p16^INK4a^‐positive cells in this proof‐of‐concept study was much higher than in vivo (Adamus et al., 2014). Other studies have suggested that epidermal stem cell function is retained throughout life, as the abundance and functional capacity of epidermal stem cells were similar between young and aged murine skin, with regard to telomere length and expression of senescent markers (Giangreco et al., 2008; Stern & Bickenbach, 2007). Similarly, keratinocytes derived from Werner syndrome patients do not show evidence of premature senescence when cultured in vitro (Ibrahim et al., 2016).

Although few differences in epidermal stem cells have been observed with age, both human and murine studies have observed that the proportion of transit‐amplifying cells is higher in the aged epidermis, and as this may not be due to epidermal stem cells, it suggests that the ageing skin phenotype could be the result of cell non‐autonomous effects (Charruyer et al., 2009; Kwon et al., 2008). This is demonstrated by senescent melanocytes, which negatively affect keratinocytes via the SASP in a non‐autonomous manner (Victorelli et al., 2019). It has also been suggested that dermoepidermal cross‐talk influences epidermal ageing (Boukamp et al., 1990).

#### 4.1.2 Cellular senescence in the dermis

Within the dermis, chronic senescence is thought to contribute to skin ageing in cell‐autonomous and non‐autonomous fashions (Demaria et al., 2015). Cell‐autonomous effects include reduced proliferation and cessation of extracellular matrix synthesis, which impairs tissue homeostasis and regeneration. Cell non‐autonomous effects include the induction of a pro‐inflammatory and pro‐oxidant SASP (Demaria et al., 2015). A bystander effect of senescence has also been proposed, whereby the SASP is thought to induce a DNA damage response in neighbouring cells, and accelerates the age‐dependent increase of senescent cells within the skin (Nelson et al., 2012). This senescent bystander effect is thought to be propagated through reactive oxygen species (ROS)‐activated nuclear factor kappa‐light‐chain‐enhancer of activated B cells (NFκB) signalling (Nelson et al., 2018).

Senescent cells also exhibit impaired mitochondrial function and increased ROS production, which are thought to contribute to the maintenance of deep senescence, the bystander effect and age‐related oxidative stress (Passos et al., 2010).

### 4.2 Oxidative stress

ROS are a natural by‐product of aerobic metabolism, and physiological levels of ROS are important for homeostasis, such as the innate and adaptive immune systems, and the regulation of cellular signalling, proliferation and differentiation (Schieber & Chandel, 2014). Increased quantities of ROS cause an oxidative imbalance, and the free radical theory of ageing proposes that the age‐related structural changes in human skin are a result of cumulative oxidative damage, caused by endogenous mitochondrial dysfunction and exacerbated by environmental stressors such as UV radiation and air pollution (Harman, 1956; Kim et al., 2016).

The endogenous production of mitochondrial superoxide free radicals occurs in peroxisomes, the endoplasmic reticulum and predominantly in the mitochondria, specifically at complex I and complex III of the electron transport chain (Murphy, 2009). Approximately 1.5 to 5% of the oxygen consumed by respiration form ROS by‐products, which can cause a pro‐oxidant state and increased oxidative stress (Poljsak & Milisav, 2012). A “mitochondrial vicious cycle theory of ageing” has also been proposed whereby the chronic generation of ROS in the electron transport chain is able to induce mutations in mitochondrial DNA (mtDNA) due to its close proximity. As mtDNA codes for subunits of enzyme complexes involved in oxidative phosphorylation, these mutations are thought to cause a dysfunctional electron transport chain and initiate a self‐propagating feed‐forward loop resulting in the accumulation of free radicals (Bandy & Davison, 1990; Hiona & Leeuwenburgh, 2008; Shokolenko et al., 2009).

In addition to the intrinsic formation of ROS, UV radiation can also induce the formation of free radicals (Jurkiewicz & Buettner, 1994). UV radiation has been found to cause mutations in mtDNA, such as a common 4977 base pair deletion (Berneburg et al., 1997; Singh et al., 2018). Due to the absence of nucleotide excision repair mechanisms, UV radiation‐induced photoproducts accumulate in mtDNA with time and increased exposure to UV radiation (Birch‐Machin et al., 2013). The accumulation of mtDNA damage is thought to be a biomarker of sun exposure, and it is colloquially referred to as “sunburnt DNA” (Birch‐Machin et al., 2013).

Antioxidant defences include enzymes such as superoxide dismutase (SOD) and catalase, and non‐enzymatic antioxidants such as glutathione and vitamin C. Endogenous and exogenous antioxidants attenuate the deleterious effects of ROS to maintain redox homeostasis, and age‐related changes have been observed, such as a 70% reduction in catalase in the human dermis (Rhie et al., 2001). SOD1^−/−^ and SOD2^−/−^ mice exhibit epidermal and dermal atrophy, similar to the clinical manifestations of human skin ageing (Murakami et al., 2009).

Reactive oxygen species have been proposed to accelerate the skin ageing phenotype through the modification of biological molecules such as DNA, lipids and proteins. In particular, oxidative damage to dermal collagenous and elastin networks alters their mechanical properties and intercellular interactions (Rinnerthaler et al., 2015). ROS can also indirectly exacerbate the degradation of the dermal fibrous network through the upregulation of MMPs and increased accumulation of senescent cells (Fisher et al., 2000).

### 4.3 Remodelling of the extracellular matrix

Matrix metalloproteinases are calcium‐dependent, zinc‐containing endopeptidases that hydrolyse peptide bonds within ECM proteins such as collagen and elastin. In non‐pathological conditions, MMPs are secreted by keratinocytes and fibroblasts, and they play an important role in aspects of tissue homeostasis including wound healing, cell migration and dermal remodelling. MMP activity is regulated by the endogenous tissue inhibitors of metalloproteinases (TIMPs). TIMPs bind to MMPs in a 1:1 stoichiometry and inhibit their activity via its chelating group that binds to the catalytic zinc atom in the MMP active site (Murphy et al., 1991). The delicate balance between the degradation and synthesis of extracellular matrix is disrupted with age, which contributes to dermal atrophy.

During ageing and the induction of cellular senescence, the fibroblast secretome undergoes compositional changes, which includes an upregulation of MMPs and pro‐oxidant components (Coppe et al., 2010; Waldera Lupa et al., 2015). ROS act as secondary messengers to indirectly upregulate MMP synthesis through stress‐activated signalling cascades, such as the mitogen‐activated protein kinase (MAPK) pathway. ROS activate the intermediate ERK, JNK and p38 proteins within the MAPK pathway, which stimulate the expression of c‐Fos and c‐Jun. c‐Fos and c‐Jun associate to form the global transcription factor activator protein 1 (AP‐1), which orchestrates many transcriptional responses including the upregulation of MMPs, inhibition of the transforming growth factor beta (TGFβ) signalling pathway and decreases type I and III procollagen synthesis (Fisher et al., 1996; Fisher, 2000; Quan et al., 2005). The increase in MMPs with age is not compensated by an increase in endogenous TIMPs, and an age‐dependent reduction was observed in serially cultured fibroblasts in vitro (Millis et al., 1992).

UV radiation and age‐dependent increases in MMPs induce a chronic degradation of the dermal collagenous and elastin networks, which exacerbates skin ageing. Interstitial collagens are sequentially fragmented by collagenases, gelatinases and stromelysins. Collagenases, such as MMP‐1, ‐8 and ‐13, hydrolyse the Gly‐Ile/Leu peptide bond at the substrate recognition site, approximately three quarters from the N‐terminus to produce collagen fragments (Miller et al., 1976). The fragmented collagen can then be further degraded at exposed sites by gelatinases (MMP‐2 and ‐9) and stromelysins (MMP‐3, ‐10 and ‐11) (Birkedal‐Hansen et al., 1993). UV radiation was found to increase the activity of collagenases and gelatinases by 4.4 and 2.3 times, respectively (Fisher et al., 1997). In addition to the degradation of the collagen network, chronic UV radiation also induces a histopathological deterioration of the elastic fibre network and the accumulation of amorphous, cross‐linked elastin fragments within the dermis. This can be partly attributed to the degradation by enzymes such as MMP‐2, MMP‐7, MMP‐9, MMP‐12, MMP‐13 and MMP‐14 (Ashworth et al., 1999). UV exposure was found to increase the expression of MMP‐12 in vivo by 11.9 fold, and photoageing has been found to make tropoelastin more susceptible to enzymatic cleavage and increase elastin degradation (Chung et al., 2002; Mora Huertas et al., 2016).

The chronic degradation of the extracellular matrix impairs the structural integrity and biomechanical properties of the dermis. The fragmented networks exert insufficient mechanical tension on the dermal fibroblasts, which causes them to collapse with age (Fisher et al., 2008). Collapsed fibroblasts synthesise low levels of extracellular matrix and secrete high quantities of degradative enzymes, which exacerbates dermal atrophy in a self‐perpetuating manner (Cole et al., 2018; Fisher et al., 2008).

### 4.4 Advanced glycation end products

AGEs are a heterogeneous group of post‐translational modifications that accumulate in the skin with age, with an increase of approximately 33% between the ages of 20 and 85 (Dyer et al., 1993). The deposition of AGEs occurs during intrinsic ageing, and it is exacerbated by environmental factors including UV radiation, smoking and dietary habits (Goldberg et al., 2004; Jeanmaire et al., 2001; Nicholl et al., 1998). AGEs are formed by sequential non‐enzymatic Maillard reactions between electrophilic carbonyl groups on reducing sugars and free amino groups on biological molecules such as proteins, lipids and histones to form a non‐stable Schiff base and Amadori products, which undergo further oxidation to form AGEs (Maillard, 1912). These glycoxidation products include *N* ^ε^‐(carboxymethyl)lysine (CML), *N* ^ε^‐(carboxyethyl)lysine (CEL) and pentosidine.

Dermal ECM proteins such as elastin and collagen are particularly susceptible to the accumulation of AGEs due to their remarkably long half‐lives of approximately 74 and 15 years, respectively (Shapiro et al., 1991; Verzijl et al., 2000). AGEs such as CML, CEL and pentosidine were found to increase linearly with age in dermal collagen (Verzijl et al., 2000). AGEs form intermolecular crosslinks between adjacent collagen fibres and modify their mechanical and functional properties; causing increased stiffness, disruption of integrin‐mediated intercellular interactions and impairing homeostatic ECM turnover (Avery & Bailey, 2006; Paul & Bailey, 1999). In addition to altering the structural properties of proteins, AGEs also bind to the receptor for AGEs (RAGE), which is a transmembrane, multi‐ligand receptor expressed in many cell types including keratinocytes and fibroblasts (Lohwasser et al., 2006). The binding of ligands to RAGE activates multiple signalling pathways such as MAPK and NF‐κB involved in inflammation, immune responses and apoptosis (Bierhaus et al., 2005). RAGE‐induced activation of NF‐κB sustains its expression through the induction of feed‐forward loops (Li & Schmidt, 1997).

The prevalence of AGEs has been demonstrated to increase with both intrinsic and extrinsic ageing, and UV radiation has been demonstrated to enhance AGE deposition in an in vitro model (Jeanmaire et al., 2001). AGEs such as CML predominantly accumulate in the amorphous, elastotic areas of photoaged skin (Mizutari et al., 1997). CML‐modified elastin has been demonstrated to exhibit decreased elasticity, increased aggregation and resistance to neutrophil elastase in vitro, which is thought to exacerbate the solar elastosis phenotype (Yoshinaga et al., 2012).

## 5 INVESTIGATING SKIN AGEING IN THE LABORATORY

As human skin ageing is complex, the use of an appropriate research platform is required. Traditionally, experimental ageing research has been conducted using either two‐dimensional (2D) in vitro assays or animal models; however, organotypic three‐dimensional (3D) models provide a more physiologically relevant, innovative approach to investigate biological mechanisms and evaluate new cosmetic interventions.

### 5.1 2D cell culture

Conventional 2D cell culture involves culturing homogeneous monolayers of ageing cells on a supraphysiologically stiff plastic substrate. 2D cell culture has been valuable in ageing research, for example, in the identification of the ageing fibroblast secretome (Waldera Lupa et al., 2015). Although this method is widely accepted, cells cultured in 2D adopt non‐physiological characteristics such as a flattened morphology, constrained apical‐basal polarity and restricted cell interactions, which ultimately affect cellular behaviour and response to external stimuli (Baker & Chen, 2012). In addition, the artificial culture conditions do not reflect the complex microenvironment in vivo. Human skin has a differentiated, stratified and keratinised structure, composed of heterogeneous cell populations that interact both homotypically and heterotypically. This complex multi‐layered and multicellular structure cannot be successfully recreated in vitro using 2D cell culture.

### 5.2 Animal models

Animal models have provided many insights into the fundamental understanding of ageing. Lifespan studies using invertebrate animals such as *Caenorhabditis elegans* have provided evidence of the ageing‐associated molecular pathways (Fontana et al., 2010). Mammalian models, such as mice, are more genetically similar to humans, and they are thought to experience a similar age‐related physiological decline. Mice have a relatively short lifespan; with a median of 24–28 months, and they can be genetically manipulated to study mechanisms of ageing such as cellular senescence (Baker et al., 2016). Early understandings of the skin ageing phenotype were confirmed using mammalian models, such as the decreased synthesis and increased degradation of collagen (Mays et al., 1991). Although mouse models have been used widely in ageing research, there are anatomical disparities between murine and human skin such as hair follicle distribution, epidermal thickness and presence of rête ridges (Wong et al., 2011). In addition to these structural differences, European Union legislation prohibits the use of animals for cosmetic research and initiatives such as the National Centre for the Replacement, Refinement and Reduction of Animals in Research aim to develop in vitro alternatives, such as skin equivalents (European Union, 2009).

### 5.3 3D bioengineered models

Due to the limitations of 2D cell culture and animal research, there is a significant demand for an alternative platform to conduct skin ageing research. Whilst ex vivo skin models are available, in vitro constructs overcome the heterogeneity and limited tissue availability. Bioengineered skin models recreate the complexity of human skin in vitro. Unlike 2D cell culture, fibroblasts grow in three‐dimensions within an organised extracellular matrix, which provides dynamic reciprocity and a more physiologically relevant microenvironment. The robust dermal compartment supports the development of a differentiated, stratified and keratinised epidermis, with spatiotemporal cellular communication and barrier properties. These advanced tissue constructs have versatile applications in fundamental ageing research, disease modelling and the screening of actives for pharmaceuticals.

## 6 RECENT ADVANCES IN THE DEVELOPMENT OF BIOENGINEERED AGEING SKIN CONSTRUCTS

The development of ageing skin models in vitro is a challenge, as ageing is a multifactorial and cumulative process that occurs throughout the lifetime of an individual. This complex phenomenon is difficult to replicate within a short amount of time, and there are many factors to consider including intrinsic and extrinsic influences, the underpinning molecular mechanisms of skin ageing and the recreation of an ageing phenotype in vitro. Several tissue engineering strategies have been applied to generate ageing skin in vitro; including the extended culture of a skin equivalent to simulate intrinsic ageing, the incorporation of cells from ageing individuals or senescent cells, the introduction of advanced glycation end products into the dermal compartment and exposure to environmental stressors (Table 1).

**TABLE 1:** Recent advances in the development of ageing skin and anti‐ageing interventions

Aspect of ageing	Composition of skin equivalent		Total culture time	Features of the ageing phenotype	Anti‐ageing interventions	References
	Cell source	Biomaterials				
Intrinsic ageing	Primary young fibroblasts and keratinocytes	Collagen‐glycosaminoglycan‐chitosan matrix	Up to 120 days	Decreased epidermal thickness Decreased keratinocyte proliferation Decreased terminal differentiation markers Duplication of lamina densa Increased senescent markers	–	Dos Santos et al. (2015)
Use of senescent cells	Primary early and late passage fibroblasts and keratinocytes	Polyester permeable membrane	38 days	No change in epidermal thickness No change in keratinocyte proliferation No change in basement membrane formation Decreased dermal thickness Increased MMP‐1 secretion	–	Janson et al. (2013)
	MMC‐treated primary fibroblasts and keratinocytes	Collagen‐glycosaminoglycan‐chitosan matrix	37 days	Decreased filaggrin expression Decreased extracellular matrix synthesis Decreased number of fibroblasts Increased MMP‐1 secretion Increased senescent markers	–	Diekmann et al. (2016)
	Stress‐induced senescent fibroblasts and keratinocytes	Rat tail collagen I matrix	17 days	Decreased epidermal thickness	Pre‐treatment of senescent fibroblasts with a *Solidago virgaurea* extract increased epidermal thickness	Lämmermann et al. (2018)
	Primary neonatal keratinocytes and UV‐induced senescent melanocytes (MelanoDerm)	Polycarbonate membrane (MelanoDerm)	Up to 21 days	Decreased epidermal thickness Decreased keratinocyte proliferation Increased senescent keratinocytes	Treatment with ABT‐737 and mitoQ decreased keratinocyte senescence and rescued epidermal atrophy	Victorelli et al. (2019)
	Stress‐induced senescent fibroblasts, and keratinocytes	Collagen matrix	24 days	Decreased epidermal thickness Increased keratinocyte proliferation Decreased terminal differentiation markers Partial impairment of skin barrier function	–	Weinmüllner et al. (2020)
Use of cells from ageing individuals	Primary young and aged fibroblasts and keratinocytes	Collagen‐glycosaminoglycan‐chitosan matrix	Up to 60 days	Decreased epidermal thickness Decreased keratinocyte proliferation Decreased epidermal organisation Decreased extracellular matrix synthesis	Treatment with a nutrient complex throughout the culture period increased keratinocyte proliferation and synthesis of extracellular matrix proteins	Lacroix et al. (2007)
	Primary adult or photoaged fibroblasts, keratinocytes and melanocytes	Bovine type I collagen matrix	18 days	Adult fibroblasts decreased pigmentation Photoaged fibroblasts increased pigmentation	–	Duval et al. (2014)
	Primary adult or ageing fibroblasts and keratinocytes	Collagen I matrix	22 days	Decreased epidermal thickness Decreased extracellular matrix synthesis Increased MMP‐1 secretion	–	Hausmann et al. (2019)
Use of advanced glycation end products	Primary human fibroblasts and keratinocytes	Glycated rat tail collagen type I matrix	14 days	Keratinocyte cytoplasm vacuolisation Differential epidermal junction expression Decreased dermal thickness Altered extracellular matrix fibre organisation	Pre‐treatment of the glycated dermis with aminoguanidine attenuated keratinocyte cytoplasm vacuolisation and increased dermal thickness	Pennacchi et al. (2015)
	Primary human fibroblasts and keratinocytes	CML, CEL, MG‐H1 or pentosidine modified collagen matrix	18 days	No change in epidermal thickness Differential changes to MMPs, basement membrane and IL‐6 depending on the AGE	–	Pageon et al. (2015)
	Primary human fibroblasts, keratinocytes and endothelial cells. Sensory neurons from murine dorsal root ganglion	Glycated collagen‐chitosan matrix	44 days	No change in epidermal thickness No change in keratinocyte proliferation Decreased terminal differentiation markers	Treatment with aminoguanidine during the culture period prevented glycation and negative effects on epidermal differentiation. Treatment with alagebrium did not decrease glycation, but attenuated changes in epidermal differentiation	Cadau et al. (2015)
	Primary fibroblasts and keratinocytes (EpiDermFT)	Polycarbonate membrane (EpiDermFT)	3 days	No change in epidermal thickness Decreased extracellular matrix synthesis Decreased terminal differentiation markers Increase in some inflammatory markers	Pre‐treatment with aminoguanidine prevented glycation and attenuated the increase in pro‐inflammatory cytokines	Lee et al. (2017)
Photoageing	Primary fibroblasts and keratinocytes	Bovine type I collagen matrix	18 days	ROS production DNA damage Fibroblast apoptosis	–	Marionnet et al. (2014)
	Primary fibroblasts and keratinocytes	Gelatin microcarrier	63 days	Decreased keratinocyte proliferation ROS production Increased MMP‐1 secretion	Pre‐treatment with retinoic acid increased keratinocyte proliferation and decreased ROS production and MMP secretion	Casale et al. (2018)

### 6.1 Simulating intrinsic ageing in vitro

As ageing is a lifelong process that takes decades to achieve in vivo, it was hypothesised that the extended culture of a skin equivalent would simulate intrinsic ageing in vitro (Dos Santos et al., 2015). After 120 days in culture, the skin equivalents exhibited many aspects of the ageing phenotype including epidermal atrophy, decreased proliferative potential of basal keratinocytes, increased senescence and reduced epidermal differentiation markers such as loricrin, filaggrin, involucrin and transglutaminase (Dos Santos et al., 2015). Age‐related alterations in the microanatomy of the basement membrane were also observed, such as duplication of the lamina densa (Dos Santos et al., 2015). As the ageing phenotype is achieved over time in culture, this model provides an opportunity to test actives that delay the ageing phenotype; however, the long culture time is undesirable for high‐throughput screening. An alternative, time‐effective strategy of bioengineering ageing skin is to incorporate senescent or ageing cells within the skin constructs.

### 6.2 Incorporating senescent cells within organotypic skin models

Bioengineered skin models provide a flexible, customised platform, with the ability to incorporate senescent or ageing cell types to recapitulate ageing skin in vitro, and investigate the underlying mechanisms of skin ageing. It is currently unclear how reciprocal interactions between the epidermis and dermis affect skin ageing; however, it has been hypothesised that epidermal‐dermal intercellular cross‐talk, through direct contact or soluble factors, affects epidermal morphology. Tissue engineering provides a valuable strategy to study epidermal‐dermal interactions within a physiologically relevant tissue model. Cellular senescence has been identified as one of the nine hallmarks of ageing, as previously discussed herein, and senescent cells accumulate in the skin with age. Ageing skin models have been developed that include senescent fibroblasts produced by serial replication, mitomycin C (MMC) or oxidative stress.

Due to their limited proliferative lifespan in vitro, serially passaged fibroblasts have been used as a model for cellular ageing. The incorporation of serially passaged fibroblasts within the dermal compartment did not affect epidermal morphogenesis or basement membrane formation; however, a thinner dermis correlated with augmented MMP‐1 secretion was observed (Janson et al., 2013). Replicatively senescent fibroblasts are widely used as a model of senescence in vitro; however, it takes a long time to reach their finite replication capacity, which would not be desirable for the routine high‐throughput generation of aged skin equivalents.

As a more rapid alternative to using replicatively senescent fibroblasts, senescence can be induced prematurely using a number of methods including oncogenic manipulation, oxidative stress and irradiation such as UV or X‐ray (Toussaint et al., 2002). Drug‐induced accelerated senescence can be applied in culture using the DNA alkylating agent MMC, which causes an enlarged morphology, β‐galactosidase expression and cell‐cycle arrest, recapitulative of the senescence phenotype (Alili et al., 2014). An aged in vitro model has been developed using this technology, by co‐culturing drug‐induced accelerated senescent fibroblasts and keratinocytes within a skin equivalent (Diekmann et al., 2016). When incorporated within a collagen‐glycosaminoglycan‐chitosan matrix, the fibroblasts exhibited an age‐related phenotype with reduced proliferation, decreased synthesis of extracellular matrix proteins and increased secretion of degradative enzymes (Diekmann et al., 2016). The senescent dermis did not affect epidermal thickness; however, some age‐related changes were observed such as decreased filaggrin expression, which is indicative of a deficient skin barrier (Diekmann et al., 2016).

An alternative method of inducing premature senescence is to use oxidative stress agents such as hydrogen peroxide (Toussaint et al., 2002). An ageing skin model was recently developed, by co‐culturing oxidative stress‐induced senescent fibroblasts within a collagen matrix, with keratinocytes (Lämmermann et al., 2018). The senescent fibroblasts induced epidermal atrophy, which correlates with epidermal ageing in vivo, and supports the importance of epidermal‐dermal interactions during ageing (Lämmermann et al., 2018). The changes in epidermal morphology could be ameliorated when senescent fibroblasts were pre‐treated with an extract from *Solidago virgaurea*, which is thought to exhibit anti‐inflammatory and weak senolytic properties (Lämmermann et al., 2018).

Many ageing models use a fully senescent dermis; however, this is an overestimate as only 20% to 60% of fibroblasts within an ageing dermis in vivo are thought to be senescent (Herbig et al., 2006; Lewis et al., 2011). To generate a more physiologically relevant ageing skin model, a recent study incorporated different ratios of senescent and non‐senescent fibroblasts within the dermal compartment of an ageing model (Weinmüllner et al., 2020). An increased proportion of senescent cells induced an age‐related phenotype including epidermal atrophy, a decrease in terminal differentiation markers, and partial impairment of the skin barrier (Weinmüllner et al., 2020).

Features of the ageing phenotype have been recreated in vitro using senescent fibroblasts; however, some differential effects were observed between these ageing skin models, such as changes in epidermal thickness (Diekmann et al., 2016; Janson et al., 2013; Lämmermann et al., 2018; Weinmüllner et al., 2020). These differences may be attributed to a number of factors such as the methods of senescence induction, the protocols used to generate skin equivalents or altered fibroblast behaviour in different dermal matrices.

In addition to full‐thickness skin models incorporating senescent fibroblasts, senescent melanocytes have also been used in an epidermal model. The presence of senescent melanocytes induced senescence and reduced proliferation in neighbouring keratinocytes, which resulted in decreased epidermal thickness. This ageing phenotype could be rescued by senolytic treatment or treatment with mitoQ, which targets mitochondrial‐generated ROS (Victorelli et al., 2019).

### 6.3 Incorporating cells from ageing donors within organotypic skin models

Many ageing skin models have been generated using senescent cells, however, cellular senescence only portrays one aspect of ageing, and it is one of nine contributors to physiological ageing in human skin (Lopez‐Otin et al., 2013). Some differences have been observed between cells obtained from ageing donors and senescent cells such as the composition of their secretome; however, research in this area is ongoing (Waldera Lupa et al., 2015). Due to these discrepancies, ageing cells are thought to be more physiologically relevant, and they have been used to generate ageing skin models.

An ageing skin construct was generated by co‐culturing ageing fibroblasts within a collagen‐glycosaminoglycan‐chitosan matrix, with young keratinocytes (Lacroix et al., 2007). Compared to skin models generated with young fibroblasts, the ageing fibroblasts induced epidermal atrophy, decreased keratinocyte proliferation and reduced organisation of the epidermal strata (Lacroix et al., 2007). Fibroblast age had a significant impact on epidermal morphology; however, some of these effects could be ameliorated by supplementation with antioxidant ingredients (Lacroix et al., 2007). A similar study demonstrated that the incorporation of ageing fibroblasts within the dermal compartment of a skin equivalent induced an ageing phenotype with epidermal thinning, a reduction in extracellular matrix synthesis and augmentation of degradative enzymes (Hausmann et al., 2019).

Many current ageing skin models have investigated the impact of an ageing dermis on the epidermis, by co‐culturing ageing or senescent fibroblasts with keratinocytes. Dermal fibroblasts are also speculated to affect additional cell types in human skin, such as melanocytes. Adult fibroblasts have been shown to decrease pigmentation whereas photoaged fibroblasts increase pigmentation, which demonstrates differences between intrinsic and extrinsic ageing on the epidermal phenotype (Duval et al., 2014).

### 6.4 Incorporation of advanced glycation end products

As discussed previously, AGEs such as CML, CEL and pentosidine accumulate in the skin with age and accelerate the ageing phenotype. Several groups have recently developed ageing models by incorporating AGEs into the dermal compartment, which demonstrate characteristic changes in ageing biomarkers (Cadau et al., 2015; Lee et al., 2017; Pageon et al., 2015; Pennacchi et al., 2015). Human skin equivalents provide a platform to further investigate the impact of AGEs on the ageing dermal phenotype, and the influence on epidermal morphology.

Glycation of collagen hydrogels through incubation with sodium glyoxylate and sodium cyanoborohydride induced the expression of CML within the dermal compartment, which disrupted the extracellular matrix organisation (Pennacchi et al., 2015). A glycated dermis also exerted significant effects on the epidermis, such as vacuolated keratinocytes and differential expression of intercellular junctions (Pennacchi et al., 2015). This study provided a proof‐of‐concept that AGEs affect epidermal and dermal morphology during ageing; however, only one type of AGE was induced in the glycated dermis, whereas many different AGEs accumulate in the ageing dermis in vivo.

To investigate the impact of different AGEs, full‐thickness skin equivalents were generated using modified collagen hydrogels containing either CML, CEL, pentosidine or methylglyoxal hydroimidazolone (MG‐H1) (Pageon et al., 2015). The incorporation of different AGEs exerted differential effects on the dermis, with regard to collagen synthesis and the secretion of MMPs (Pageon et al., 2015). The combinatorial effect of these AGEs is thought to be both complementary, for example in the upregulation of procollagen I, and antagonistic, as CML and CEL upregulate and downregulate TIMP1, respectively (Pageon et al., 2015). As the ageing human dermis contains a heterogeneous combination of different AGEs, it is thought that the individual AGEs may counterbalance each other to minimise their detrimental effect.

A major target of glycation during ageing is the microvasculature and sensory network, therefore to further investigate the biological effect of AGEs, a more advanced glycated in vitro skin model was developed, which integrates endothelial cells and neurons (Cadau et al., 2015). Similarly to other studies, AGEs decreased epidermal organisation and differentiation and had a deleterious effect on the nervous networks (Cadau et al., 2015).

Another novel ageing skin model studied glycation‐associated skin ageing using a commercially available skin equivalent. This study treated the EpiDermFT^TM^ full‐thickness skin equivalent with repeated glyoxal applications, which increased levels of CML within the epidermal and dermal compartments (Lee et al., 2017). Glycation of the skin equivalent induced several age‐related changes including an impaired skin barrier, upregulation of pro‐inflammatory cytokines and reduced synthesis of extracellular matrix proteins (Lee et al., 2017). The adaptation of a commercially available skin equivalent to study ageing is valuable as it provides a platform for product development and overcomes the requirement for highly technical methodologies and specialised equipment.

Despite only modelling one aspect of ageing skin, most of these models used a well‐characterised AGE inhibitor, aminoguanidine, which was demonstrated to consistently suppress the age‐related phenotype. These ageing skin models could also be used to screen additional anti‐glycation compounds, such as niacin and the rutin flavonoid (Abdullah et al., 2017; Nagasawa et al., 2003).

### 6.5 Simulating extrinsic ageing and photoageing in vitro

As the skin is at the interface with the environment, it is exposed to external stressors such as UV radiation and air pollution, which contribute to extrinsic skin ageing. In contrast to incorporating ageing cells or glycation to tailor skin equivalents for ageing studies, the contribution of environmental exposure to the ageing phenotype can be assessed in vitro by exposing skin models to exogenous stressors such as UV radiation (Casale et al., 2018; Marionnet et al., 2014).

Solar UV radiation contributes to the electromagnetic spectrum that reaches the Earth's surface, and it is categorised according to wavelength: UVA (315–400 nm), UVB (280–315 nm) and UVC (100–280 nm). UVC is associated with biological mutagenic and carcinogenic effects, but is absorbed by the atmospheric ozone and does not reach the Earth's surface. UVB is absorbed in the epidermis and causes acute photodamage including keratinocyte apoptosis and DNA damage via the formation of cyclobutane pyrimidine dimers and 6–4 photoproducts (Mouret et al., 2006). The longer wavelength of UVA radiation allows it to penetrate into the dermis, and it is thought to be responsible for the majority of chronic photoageing (Battie & Verschoore, 2012; Imokawa & Ishida, 2015).

Human skin equivalents have been applied to investigate the impact of UV radiation on the structure of human skin, and its contribution to photodamage and photoageing (Casale et al., 2018; Marionnet et al., 2014). Irradiation of skin equivalents with UVA was found to induce characteristic features of skin ageing, including increased oxidative stress, decreased proliferative capacity of keratinocytes and degradation of the dermal extracellular matrix (Casale et al., 2018; Marionnet et al., 2014). These studies demonstrate the use of skin equivalents to recapitulate features of extrinsic ageing in vitro.

## 7 NEXT GENERATION AGEING SKIN MODELS

**FIGURE 4:**
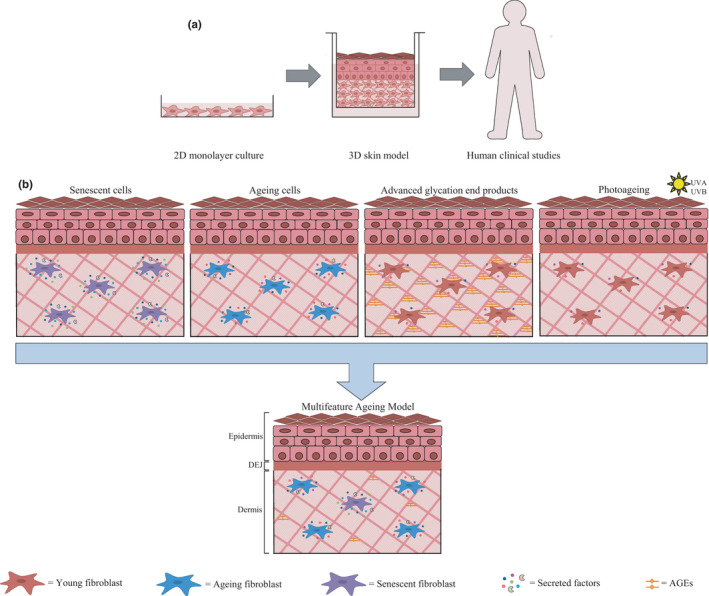
Bioengineering the next generation of ageing skin models in vitro. (a) 3D skin models provide a more predictive platform for fundamental and translational research, to bridge the gap between 2D cell culture and human clinical studies. (b) Advanced ageing skin models could incorporate several molecular mechanisms to more accurately model the complexity of human skin ageing

Current ageing skin models represent different molecular mechanisms of skin ageing, which provides a combination of tools to elucidate their relative contribution to the ageing phenotype, and identify preventative materials. However, as skin ageing is complex and multifactorial, future advancements could include in vitro skin equivalents that encompass several mechanisms, as a more accurate recapitulation of the ageing phenotype (Figure 4). This would also enable the cross‐talk between different mechanisms to be researched further, for example the impact of ageing or senescent fibroblasts on AGE accumulation, and conversely, the impact of AGEs on senescence induction.

The next generation of ageing skin models could also incorporate additional cell types. Ageing full‐thickness skin equivalents are currently bioengineered using young keratinocytes and ageing dermal compartments; however, future work could include the co‐culture of both ageing fibroblasts and ageing keratinocytes. We hypothesise that the co‐culture of these two ageing cell types could provide a more physiologically relevant ageing skin equivalent. In addition, increasing the model complexity through the inclusion of additional cell types such as melanocytes and immune cells including Langerhans cells, macrophages and dendritic cells would recreate more complex spatiotemporal interactions between different cell types during skin ageing. It would also provide a platform to understand how different cell populations interact during skin ageing, for example, it has been demonstrated that photoaged fibroblasts increase skin pigmentation, and senescent melanocytes affect basal keratinocyte proliferation and induce epidermal atrophy (Duval et al., 2014; Victorelli et al., 2019). The presence of immune cells is particularly important as skin ageing is associated with chronic, low‐grade inflammation, termed “inflammaging,” which is thought to accelerate the skin ageing phenotype (Zhuang & Lyga, 2014).

## 8 VALIDATION AND APPLICATION OF AGEING SKIN MODELS

There have been many recent advances in the development of ageing skin equivalents that recapitulate key aspects of skin ageing. The current gold standard for assessing an ageing phenotype is investigating fundamental age‐related changes such as epidermal atrophy, decreased keratinocyte proliferation and decreased dermal extracellular matrix using techniques such as immunofluorescence staining, gene expression and protein analysis. Whilst this is widely accepted, a more in‐depth characterisation would provide an indication of how accurately the ageing skin models resemble the in vivo morphology.

Many challenges exist due to the heterogeneity of skin ageing; therefore, it is important to elucidate and define a universal panel of ageing biomarkers that represent epidermal and dermal ageing for the assessment of in vitro ageing models. In addition to the current gold standard assessments of skin structure, characterisation of skin functionality could include the assessment of epidermal barrier properties such as transepidermal water loss and *stratum corneum* hydration. These non‐invasive techniques would provide a direct comparison to end‐points in in vivo clinical studies. Complete validation of the ageing skin models should also include functional testing in comparison with human skin, such as measuring the response to a validated active compound.

Ageing skin models could be used to screen anti‐ageing interventions and determine the relative contribution of different molecular mechanisms to the ageing skin phenotype. Ageing skin equivalents that integrate senescent cells provide a useful platform for screening senolytics. Senolytics selectively ablate senescent cells, or target the deleterious SASP, and proof‐of‐principle studies have demonstrated that a *Solidago virgaurea* extract with weak senolytic properties improved the ageing skin model morphology, and the elimination of senescent melanocytes using the senolytic ABT‐737 rescued features such as epidermal thickness (Lämmermann et al., 2018; Victorelli et al., 2019). Treatment of ageing skin models with antioxidants also improved the epidermal organisation and increased keratinocyte proliferation (Lacroix et al., 2007). Many studies supplement the culture media with actives; however, this does not resemble the penetration of actives in vivo, as formulations are often applied to the skin surface. A recent study topically applied aminoguanidine, an anti‐glycation active, to the surface of a glycated ageing skin model and observed a reduction in CML and suppression of pro‐inflammatory cytokines (Lee et al., 2017).

## 9 CONCLUSIONS AND FUTURE DIRECTIONS

Due to the world ageing demographic, there is an expected increase in the prevalence of age‐related dermatoses. Due to fundamental anatomical differences and the recent ban on the use of animals for cosmetic testing, it is becoming increasingly necessary to develop robust organotypic models of ageing human skin (European Union, 2009; Gerber et al., 2014). These models provide a valuable tool to investigate the interconnected molecular mechanisms of skin ageing and associated diseases, and a platform for the screening of pharmaceutical and cosmetic interventions.

There have been many recent advances in bioengineered ageing skin models using different approaches, including the incorporation of ageing or senescent cells, advanced glycation end products or exposure to extrinsic stressors. Future advancements of ageing skin models include the use of additional cell types such as melanocytes and immune cells, and the inclusion of several molecular mechanisms to model the ageing skin phenotype more accurately.

The gold standard assessment of ageing skin models also needs to be re‐evaluated, to determine how accurately they resemble in vivo skin ageing, through in‐depth analytical techniques and comprehensive structural and functional validation, in comparison with real ageing human skin. Multidisciplinary collaborations between cellular biologists, dermatologists and bioengineers are essential to the future advancement of this exciting field, which has the potential to revolutionise the next generation of healthcare product development.

## CONFLICT OF INTEREST

SP acts as a consultant for Reprocell Europe.

## AUTHOR CONTRIBUTIONS

LC produced the initial draft of the review. TD, RT, RI, CB, TvZ and SP reviewed the article, contributed to the content and agreed the final version.

## Data Availability

Not applicable.
